# Evaluation of Prescription Medication Sharing Among Adults in South Korea: A Cross-Sectional Survey

**DOI:** 10.3389/fphar.2022.773454

**Published:** 2022-02-04

**Authors:** Seulki Song, Seungyeon Kim, Sangyoon Shin, Young Lee, Euni Lee

**Affiliations:** ^1^ College of Pharmacy and Research Institute of Pharmaceutical Sciences, Seoul National University, Seoul, South Korea; ^2^ Department of Pharmacy, Veterans Health Service Medical Center, Seoul, South Korea; ^3^ Transdisciplinary Department of Medicine and Advanced Technology, Seoul National University Hospital, Seoul, South Korea; ^4^ Veterans Medical Research Institute, Veterans Health Service Medical Center, Seoul, South Korea; ^5^ Department of Applied Statistics, Chung-Ang University, Seoul, South Korea

**Keywords:** medication sharing, prescription medication sharing, medication lending, medication borrowing, inappropriate medication use

## Abstract

**Background:** Prescription medication sharing is an inappropriate medication use behavior that can lead to medication errors and adverse drug events, posing a public health threat. The reported prevalence of prescription medication lending and borrowing varies by country, ranging from 6%–23% and 5%–52%, respectively. However, research on medication sharing is scant in Asian countries. Therefore, this study aimed to describe the rate of prescription medication sharing practices and investigate the associated behavioral factors, types of shared medications, and reasons for sharing among adults in South Korea.

**Methods:** A cross-sectional study was conducted using an online self-administered survey of 1,000 adults (aged 19–69 years; November 2020). A stratified sampling method was used to select survey participants from a nationwide consumer panel, which ensured a representative distribution of the Korean population by age, gender, and region. Descriptive and logistic regression analyses were used to evaluate the information related to sharing behavior.

**Results:** A total of 1,000 respondents participated in this study. The mean age of the respondents was 44.7 years (standard deviation [SD], 13.4), ranging from 20 to 69 years. The rate of medication sharing was 52.4%. The most prevalently shared medications were analgesic, antipyretic, and antimigraine medications. Prescription medications were shared mostly between family and relatives. Older age was a predictive factor for sharing analgesics. Lower educational level was a predictive factor for sharing ophthalmic medications.

**Conclusions:** Approximately one in two respondents in our study have experienced medication sharing in their lifetime. Future studies are needed to establish evidence-based strategies for patient education and improve the medication use process. Healthcare professionals should assess patients’ needs for accessing medications and be ready to educate and guide them with specific action plans. Policymakers should consider patient empowerment strategies including public education and campaigns to avoid potential adverse outcomes of medication sharing.

## Introduction

Medication sharing is defined as lending or borrowing medication (Daniel et al., 2003). Although the terms “to borrow” and “to lend” also imply returning an item to its owner, “medication sharing” in social science research is considered as the act of giving or receiving medications without returning them to the owner (Beyene et al., 2014). When the shared medication requires a prescription from healthcare providers, this phenomenon is referred to as “prescription medication sharing” (Daniel et al., 2003). The reported prevalence of prescription medication lending and borrowing varies by country, ranging from 6 to 23% and 5%–52%, respectively (Beyene et al., 2014). Prescription medication sharing is an inappropriate medication use behavior that can lead to medication errors and adverse drug events. In an aging society with many older adults having multimorbidity and polypharmacy (Golchin et al., 2015), the increased availability of medications for sharing and the vulnerability of older adults towards adverse drug events can potentially complicate the clinical consequences for individuals. Furthermore, prevalent medication sharing at the community level poses a public health threat (Goldsworthy et al., 2008).

Many studies have been conducted to investigate behaviors related to medication sharing, such as the circumstances and predictors associated with this practice. Some studies have reported that being female (Daniel et al., 2003; Goldsworthy et al., 2008); being 18–24 years of age (Petersen et al., 2008); being a non-Hispanic White, reproductive-aged female United States resident (Petersen et al., 2008); having lower income (Daniel et al., 2003); deriving health-related information from the Internet (Petersen et al., 2008); having a larger household size (Daniel et al., 2003; Petersen et al., 2008); having non-Medicare insurance (Ward et al., 2011); and having leftover medications stored at home (Beyene et al., 2019) were associated with increased medication sharing. In addition, potential reasons for sharing medication include emergency situations, inconvenience in visiting a physician, lack of trust in the physician, low accessibility to the medication, availability of medications from close acquaintances, cost saving, assisting with pain management, and maintaining good relationships with people (Markotic et al., 2017). Unintended and unexpected consequences of medication sharing caused by unsupervised and reckless medication use have also been documented, such as delayed diagnosis or treatment, incorrect perceptions of the ineffectiveness of the medication, drug interactions, adverse drug reactions, drug abuse, addiction, antibiotic resistance, and teratogenicity (Daniel et al., 2003; Goldsworthy et al., 2008; Petersen et al., 2008; Markotic et al., 2017).

Medication sharing is considered an underestimated problem in the medical field (Petersen et al., 2008; Auta et al., 2011). In South Korea, research on the status of medication sharing behavior and associated factors is scant. Therefore, this study aimed to describe the rate of prescription medication sharing practices and investigate the associated behavioral factors, types of shared medications, and reasons for medication sharing among adults in South Korea.

## Materials and Methods

### Study Design and Population

A cross-sectional study was conducted using an online self-administered survey to evaluate the experience of medication sharing among adults aged 19 years or above, excluding healthcare professionals. Participants were recruited using a commercially available consumer panel of a nationwide convenience sample operated by the Panel Marketing Interactive company, which included 1.59 million people, one of the biggest consumer panels in South Korea in 2020 (Panel Marketing Interactive, Seoul, South Korea; Kim et al., 2020). A more detailed description of the panel has been published elsewhere (Panel Marketing Interactive, 2021).

A stratified sampling scheme was used to select survey participants from the panel, which ensured that they were representative of the distribution of the national census statistics by age, gender, and region (Korean Statistical Information Service, 2019). Considering that the oldest panel from the marketing company was set at 69 years of age, and the relative difficulty in accessing the Internet among older adults, Koreans older than 70 years were not included. Participants were recruited through the company’s website or an e-mail inviting them to participate in the study, and a link was provided in the recruitment announcement (banner) to lead them to the survey screen. The survey questionnaire included four screening questions for ensuring the participant’s eligibility in terms of having non-healthcare-related jobs and other pre-determined quotas for demographic information, such as gender, age, and region. Prior to commencing the survey, all participants responded to the screening questions and those who met the eligibility criteria agreed to participate in the survey by providing informed consent. The survey was conducted between November 6 and 12 November 2020. The study protocol was approved by the Institutional Review Board (IRB) of Seoul National University (IRB No. 2009/002-012). This study has been conformed to the principles embodied in the Declaration of Helsinki.

### Questionnaire Development

The questionnaire was developed following the conceptual framework of the modified Andersen behavioral model of health, which includes the outcome of health behaviors (Andersen, 1995). The model was developed to determine why families use health services, and health behavior was considered a function of three factors: 1) predisposing characteristics such as demographics, social structure, and health beliefs; 2) enabling resources from personal/family sources or the community; and 3) perceived or evaluated needs. In this study, the variable of health behavior was replaced with medication sharing behavior. The literature on medication sharing was consulted to extract factors which were then categorized into sectors in the model. The survey questionnaire comprised three themes: 1) demographic characteristics, 2) lending experience, and 3) borrowing experience. Demographic characteristics consisted of predisposing factors including gender, age, and marital status, as well as enabling resources including educational level, type of health insurance, and area of residence. Questions regarding lending and borrowing experiences included the participants’ lifetime experience of medication sharing, types of people with whom they had shared medication, types of medications shared, and reasons for sharing. The reasons for lending or borrowing medication consisted of enabling factors and need factors. The reasons for medication sharing included severity of symptoms (Goulding et al., 2011), limited access to physicians (Goulding et al., 2011; Ward et al., 2011), urgency (Daniel et al., 2003; Goldsworthy et al., 2008; Petersen et al., 2008), medication potency (Daniel et al., 2003; Goldsworthy et al., 2008; Petersen et al., 2008), accessibility to medication (Daniel et al., 2003; Goldsworthy et al., 2008; Petersen et al., 2008; Markotic et al., 2017), altruism (Markotic et al., 2017), and having faith in the lender (Daniel et al., 2003; Goldsworthy et al., 2008; Petersen et al., 2008). The list of medications that were lent or borrowed included pain or fever relievers, intestinal medications, ophthalmic medications, antibiotics, topical corticosteroids, muscle relaxants, allergy medications, hypnotics, mood medications, contraceptives, inhalers for asthma, and chronic disease medications for hypertension, diabetes, or hyperlipidemia. The list of medications was formulated based on the literature on medication sharing (Goldsworthy et al., 2008; Petersen et al., 2008; Goulding et al., 2011; Ward et al., 2011; Beyene et al., 2014) and prevalent chronic diseases in adults (Korea Centers for Disease Control and Prevention, 2018). The medication list used in the questionnaire stated the class of the medications and explained the medications in lay terms. The questionnaire also asked participants whether the medication package insert or instructions for medication use were provided during medication sharing. The consequences of taking shared medications were also assessed as an outcome factor.

After the development of the questionnaire, a face validation was conducted with five pharmacists and five non-healthcare personnel to evaluate the appropriateness, clarity, and readability of the questions. The final version of the questionnaire comprised a total of 20 questions, including four screening questions and 16 main questions, in three sections: lending experience (6 questions), borrowing experience (6 questions), and demographic characteristics (4 questions). Not all participants responded to all 20 questions; respondents with no medication sharing experience completed a total of 10 questions: four screening questions, two questions on lending and borrowing experience (yes/no), and four questions regarding demographic characteristics. The questions about lending and borrowing experiences were in separate sections. When the respondent clicked on “yes” for the question on having lending experience, additional questions regarding their lending experience were asked consecutively. If the respondent selected “no” as their response to this question, the questionnaire skipped to the borrowing experience section. When the borrowing experience section was completed, the demographic section began.

### Sample Size

The target number of survey participants was calculated to evaluate the prevalence of medication sharing practices in South Korea. From the literature review, the average prevalence was found to be approximately 25% (Daniel et al., 2003; Goldsworthy et al., 2008; Petersen et al., 2008; Auta et al., 2011; Goulding et al., 2011; Beyene et al., 2014), and the prevalence in our study was expected to be 30%. The sample size was calculated using the G*power 3.1.9.4 program (Heinrich-Heine-Universität Düsseldorf, Düsseldorf, Germany) (Faul et al., 2007) based on a two-sided binomial test, with 90% power and 5% significance level, yielding 842 participants. Considering screening failure and participant dropout rate (15%), the target sample size of the study was determined to be 1,000.

### Statistical Analysis

Descriptive statistics were used to summarize respondents’ demographic characteristics and information related to medication sharing. Continuous variables were analyzed using student’s *t*-test, while categorical demographic variables were analyzed using Pearson’s chi-squared test or Fisher’s exact test. Medication sharing prevalence was examined by gender, age group (20–29, 30–39, 40–49, 50–59, and 60–69 years), marital status, metropolitan status, residential area, level of education, and health insurance type. Multiple logistic regression analysis was used to identify factors associated with medication sharing behavior. Subgroup analyses were performed for each medication’s lending and borrowing experience to determine the factors contributing to the sharing behavior for each medication. Statistical analyses were performed using SPSS 25 (IBM Corp, Armonk, NY) and R 4.0.1 (R Foundation for Statistical Computing, Vienna, Austria). The level of statistical significance was set at *p* < 0.05.

## Results

### Participants’ Characteristics

A total of 1,000 people participated in this study; questionnaire distribution was terminated when the target number of respondents was achieved. The mean age of the participants was 44.7 years (standard deviation [SD], 13.4), ranging from 20 to 69 years. The ratio of men to women was approximately 1:1. More than half of the respondents were married (62.9%), and most (91.4%) lived in urban areas. Over 70% of the respondents had a college degree or higher, and most of them had workplace health insurance (69.8%). The demographic characteristics are presented in detail in Table 1.

**TABLE 1:** Survey respondents’ demographic characteristics and the rates of lending or borrowing prescription medications (*n* = 1,000).

Characteristics	Number of respondents (%)*	Have lent prescription medications	Have borrowed prescription medications
		Prevalence (95% CI)	Prevalence (95% CI)
Total	1,000 (100.0)	40.6 (37.6–43.6)	43.0 (39.9–46.1)
Gender			
Female	492 (49.2)	42.1 (37.7–46.4)	42.9 (38.5–47.3)
Male	508 (50.8)	39.2 (34.9–43.4)	43.1 (38.8–47.4)
Age distribution			
20–29 years	182 (18.2)	37.9 (30.9–45.0)	42.3 (35.1–49.5)
30–39 years	187 (18.7)	36.9 (30.0–43.8)	44.4 (37.3–51.5)
40–49 years	224 (22.4)	42.9 (36.4–49.3)	45.1 (38.6–51.6)
50–59 years	232 (23.2)	44.0 (37.6–50.4)	42.2 (35.9–48.6)
60–69 years	175 (17.5)	40.0 (32.7–47.3)	40.6 (33.3–47.8)
Marital status			
Single	371 (37.1)	37.5 (32.5–42.4)	41.8 (36.8–46.8)
Married	629 (62.9)	42.4 (38.6–46.3)	43.7 (39.8–47.6)
Metropolitan status			
Urban	914 (91.4)	40.9 (37.7–44.1)	43.9 (40.7–47.1)
Rural	86 (8.6)	37.2 (27.0–47.4)	33.7 (23.7–43.7)
Residential area			
Capital	194 (19.4)	44.3 (37.3–51.3)	47.4 (40.4–54.5)
Metropolitan cities	250 (25.0)	36.4 (30.4–42.4)	41.2 (35.1–47.3)
Provinces	556 (55.6)	41.2 (37.1–45.3)	42.3 (38.2–46.4)
Level of education			
High school and below	291 (29.1)	40.9 (35.2–46.5)	43.6 (37.9–49.3)
College and above	709 (70.9)	40.5 (36.9–44.1)	42.7 (39.1–46.4)
Health insurance type			
Self-employed	239 (23.9)	41.0 (34.8–47.2)	43.1 (36.8–49.4)
Workplace	698 (69.8)	41.1 (37.5–44.8)	42.8 (39.2–46.5)
Others^a^	63 (6.3)	33.3 (21.7–45.0)	44.4 (32.2–56.7)

a Others include Medical Aid, no health insurance, or unknown.

* Percentage was calculated in column.

*CI*, confidence interval.

### Reported Experience of Lending or Borrowing Prescription Medication

Among the 1,000 respondents, 524 reported having either medication lending or borrowing experiences, while 312 reported having experienced both medication lending and borrowing. The rates of medication lending and borrowing experience were 40.6% and 43.0%, respectively. The mean age of participants who had lent and borrowed medication was 45.3 years (SD: 13.4) and 44.5 years (SD: 13.5), respectively. Table 1 presents the prevalence of medication lending and borrowing based on the respondents’ demographic characteristics.

Medications were primarily lent to or borrowed from family and relatives (86.9% for lending and 83.5% for borrowing) and friends (23.2% and 21.2%, respectively). These numbers were not mutually exclusive. The most frequent reason for both lending and borrowing was that the lender happened to have the medication the borrower needed (58.4% for lending and 45.3% for borrowing). The least frequent reason for lending was that the lender just wanted to help the borrower (7.1%), and that for borrowing was that the borrower wanted stronger medication (7.9%; Supplementary Table S1). Most of the respondents who had lent medication (91.4%) provided either a verbal explanation or an instruction sheet when they lent the medication. However, 20% of the respondents who had borrowing experience reported having received neither a verbal explanation nor an instruction sheet. Most respondents (89.4% for lending and 87.0% for borrowing) reported that the borrower’s condition improved after taking the medication.

### Prescription Medications Subject to Lending or Borrowing

The rankings of medications that had been lent or borrowed were similar. Pain relievers, including analgesic, antipyretic, and antimigraine medications, were the most frequently lent and borrowed ones (Table 2). The reported frequency of gastroduodenal ulcer medication and ophthalmic medication lending was over 20%, whereas borrowing of these medications was below 20%. Antibiotics were also shared by more than 10% of the respondents. Less than 5% of the respondents shared chronic disease medications. The details are presented in Table 2.

**TABLE 2:** Reported frequencies and types of prescription medications that were lent and borrowed.

Type of medication	Lent prescription medications (*n* = 406), No. (%*)	Borrowed prescription medications (*n* = 430), No. (%*)
Analgesics	218 (53.7)	204 (47.4)
Antipyretics	173 (42.6)	147 (34.2)
Antimigraine	100 (24.6)	91 (21.2)
Gastroduodenal ulcer medications	99 (24.4)	73 (17.0)
Ophthalmic medications	88 (21.7)	63 (14.7)
Laxatives or antidiarrheal medications	73 (18.0)	68 (15.8)
Antibiotics	65 (16.0)	55 (12.8)
Topical corticosteroids	59 (14.5)	53 (12.3)
Muscle relaxants	41 (10.1)	38 (8.8)
Allergy medications	38 (9.4)	31 (7.2)
Hypnotics	23 (5.7)	22 (5.1)
Antihypertensives	17 (4.2)	9 (2.1)
Psoriasis medications	16 (3.9)	24 (5.6)
Diabetes medications	11 (2.7)	5 (1.2)
Hyperlipidemia medications	10 (2.5)	9 (2.1)
Mood medications	7 (1.7)	1 (0.2)
Contraceptives	6 (1.5)	2 (0.5)
Inhalers for asthma	1 (0.2)	2 (0.5)
Others	19^a^ (4.7)	26^b^ (6.0)

a Others include decongestants, antitussives, expectorants, and medications for cold or weight loss.

b Others include decongestants, antitussives, expectorants, medications for cold, weight loss, hair loss, rhinitis, dental care, and nutritional supplements.

* As the respondents were asked to report their lending or borrowing experience for a list of 19 medications, the numbers are not mutually exclusive.

### Associated Factors for Prescription Medication Lending or Borrowing

There were no statistically significant differences related to lending or borrowing experiences based on participants’ demographic characteristics (Table 1). The mean age of the groups with and without medication lending experience and that of groups with and without medication borrowing experience showed no statistically significant differences (*p* = 0.208 and *p* = 0.763, respectively). However, some associated factors were determined for the sharing behavior for each medication type in the subgroup analyses. Among the prescription medications, the results for analgesics, gastroduodenal ulcer medications, ophthalmic medications, antibiotics, and hypnotics showed significant factors contributing to lending or borrowing behavior.

Respondents aged 60 years or older were more likely to lend analgesics than those aged 20–29 years (odds ratio [OR], 2.47; 95% confidence interval [CI], 1.06–5.75). Respondents aged 40–49 years (OR, 3.28; 95% CI, 1.56–6.92) and 60 years and older (OR, 3.79; 95% CI, 1.63–8.81) were more likely to borrow analgesics than respondents aged 20–29 years (Table 3).

**TABLE 3:** Factors associated with lending or borrowing experience for analgesics.

Characteristics	Have lent analgesics (*n* = 218) OR (95% CI)^a^	Have borrowed analgesics (*n* = 204) OR (95% CI)^a^
Gender		
Female	1.0	1.0
Male	1.13 (0.74–1.70)	1.17 (0.78–1.77)
Age distribution		
20–29 years	1.0	1.0
30–39 years	1.45 (0.69–3.03)	1.76 (0.84–3.69)
40–49 years	1.61 (0.78–3.32)	3.28 (1.56–6.92)^†^
50–59 years	1.63 (0.77–3.48)	2.09 (0.96–4.53)
60–69 years	2.47 (1.06–5.75)*	3.79 (1.63–8.81)^†^
Marital status		
Single	1.0	1.0
Married	1.02 (0.58–1.78)	0.90 (0.52–1.55)
Residential area		
Capital	1.0	1.0
Metropolitan cities	0.94 (0.51–1.72)	0.44 (0.24–0.80)^†^
Provinces	0.77 (0.46–1.30)	0.57 (0.34–0.95)*
Level of education		
High school and below	1.0	1.0
College and above	1.03 (0.64–1.65)	1.02 (0.63–1.64)
Health insurance type		
Self-employed	1.0	1.0
Workplace	1.43 (0.88–2.34)	1.23 (0.75–2.03)
Others^b^	1.65 (0.61–4.47)	1.54 (0.63–3.80)

a Multiple logistic regression analysis.

b Others include Medical Aid, no health insurance, or unknown.

*p< 0.05

† *p*< 0.01.

*CI*, confidence interval; *OR*, odds ratio.

Respondents aged 50 years and older were more likely to lend or borrow gastroduodenal ulcer medications than respondents aged 20–29 years (Supplementary Tables S2, 3). Regarding ophthalmic medications, respondents who had an educational level of college and above were less likely to lend these medications than those with an educational level of high school and below (OR, 0.54; 95% CI, 0.31–0.93; Supplementary Table S2). In the case of antibiotics, respondents in their 30s (OR, 4.92; 95% CI, 1.68–14.44), 50s (OR, 3.36; 95% CI, 1.05–10.67), and 60s (OR, 5.65; 95% CI, 1.65–19.29) were more inclined to lend antibiotics than those in their 20s; married people were less inclined to lend antibiotics than those who were single (OR, 0.40; 95% CI, 0.19–0.81; Supplementary Table S2). Respondents aged 30–39 years tended to borrow more antibiotics than those aged 20–29 years (OR, 3.64; 95% CI, 1.12–11.79; Supplementary Table S3).

Hypnotics tended to be borrowed less by male respondents than female respondents (OR, 0.25, 95% CI, 0.09–0.69). They were borrowed more by respondents in their 30s (OR, 15.89; 95% CI, 1.70–148.34), 40s (OR, 10.87; 95% CI, 1.08–108.99), and 60s (OR, 50.87; 95% CI, 4.34–595.88) than those in their 20s. Married people were less likely to borrow hypnotics than those who were single (OR, 0.14; 95% CI, 0.04–0.47) (Supplementary Table S3).

## Discussion

To our knowledge, our study is the first of its kind to report the prevalence of prescription medication sharing, the most frequently shared medications, and their associated factors using a relatively large sample in South Korea. We believe it provided comprehensive overview of medication sharing behavior among Korean adults. Pharmacotherapy is the mainstay modality for treating many clinical conditions (Upadhyay et al., 2018). Therefore, access to medication and its appropriate use is one of the most important healthcare agendas (World Health Organization, 2017). Timely and proper access to medication has been a critical issue for improving health outcomes for patients, clinicians, and policymakers not only for enhancing legitimate access to prescription medications but also for avoiding the misuse or illicit use of medications (World Health Organization, 2021). Although many studies have addressed ways to improve clinical outcomes with appropriate use of pharmacotherapy worldwide (Whelton et al., 2018), studies on medication sharing have been conducted by researchers from limited number of countries. The majority of the published studies on the topic were from countries in Europe, America, and Oceania (Daniel et al., 2003; Sorensen et al., 2003; Goldsworthy et al., 2008; Petersen et al., 2008; Goulding et al., 2011; Ward et al., 2011; Beyene et al., 2014; Dohn and Pilkington, 2014; Markotic et al., 2017; Markotic et al., 2018; Beyene et al., 2019a; Beyene et al., 2019b; Beyene et al., 2019c), very few studies were reported from African countries and Middle East (Yousif, 2002; Jassim, 2010; Sharif et al., 2010; Auta et al., 2011; Kheir et al., 2011; Ocan et al., 2015; Asmelashe Gelayee and Binega, 2017; Mostafa-Hedeab, 2018; Obol et al., 2018; Torres et al., 2019; Alhomoud, 2020), even fewer studies were conducted from countries by researchers in Asia including Korea (Ali et al., 2010; Baber et al., 2017; Atif et al., 2019). While a few studies mentioned presence of medication sharing as a part of medication misuse (Jeong, 2017; Park and Jang, 2018), no studies were found from a literature search using keywords of medication sharing from the Research Information Sharing Service (RISS), a Korean research data search engine. Therefore, our study can serve as a point of reference highlighting overall practice of prescription medication sharing among Korean adults.

Our findings indicate that one in two Korean adults share prescription medications. The estimated prevalence is similar to that reported in Nigeria (52.7%) (Auta et al., 2011), but our observed prevalence rate was mostly higher than the rates observed in the United States and Ireland, with the reported rates of lending or borrowing ranging between 13.4% and 34.1%, respectively (Daniel et al., 2003; Goldsworthy et al., 2008; Petersen et al., 2008; Goulding et al., 2011; Ward et al., 2011; Beyene et al., 2014). The exact reasons why the rates of medication sharing differ between countries remain unclear; however, the potential reasons could be related, in part, to differences in enabling resources like access to healthcare services and prescription medication health insurance coverage, as well as to the diversity of health beliefs or culture in each society related to sharing practices (Beyene et al., 2014). Reports from the literature also indicate enabling resources like presence of leftover medications as predictors of medication sharing (Beyene et al., 2019a). In fact, in 2018, a Korean government report estimated that the cost of unused prescribed medications was 218 billion Won (USD 192,579,505 as of 2021) in South Korea (Health Insurance Review and Assessment Service, 2019). We believe that leftover medications should be explored as the potential resources for medication sharing in the future, because the most common reason for medication sharing in our study was accessibility to medication from others, especially from family and relatives.

Our findings showed that analgesics and gastroduodenal ulcer medications were the most frequently shared medications. Based on reports from the Health Insurance Review and Assessment Service in South Korea, these ranked in the top five medications that have been most frequently billed over the past 3 years (Healthcare Bigdata Hub. Health Insurance Review and Assessment Service, 2021). Further, the tendency of borrowing behavior for hypnotics was higher in women and people with older age when we explored the association between medication sharing and predisposing factors. This can be attributed to the reported strong predictors of insomnia medication use by women and older adults (Bertisch et al., 2014; Wilson et al., 2019). The increased medication usage can be linked to the possibility of medication sharing.

Studies from many countries have reported the frequent sharing of analgesics (20%–61%) (Sorensen et al., 2003; Petersen et al., 2008; Auta et al., 2011; Markotic et al., 2018). Moreover, as the prevalence of using prescription pain medications increases with increasing age (Hales et al., 2020), sharing of analgesics may also be affected by increasing age. More attention should be paid to the potential risks of analgesic-sharing, such as missed opportunities for timely diagnosis by physicians and adverse drug reactions, including hypertension and nephrotoxicity in frail older adults (Petersen et al., 2008; Beyene et al., 2014; Wongrakpanich, et al., 2018). Along with analgesics, antibiotics are a well-known shared medication (Beyene et al., 2014). Sharing of antibiotics can complicate the efficacy and safety of the medication use process not only at the personal level but also at the public health level due to antimicrobial resistance (World Health Organization, 2020).

The prevalent sharing of analgesics and antibiotics is a major public health concern. However, less frequently shared medications with reported serious adverse consequences should also be highlighted. Ophthalmic medications may include, but are not limited to, artificial tears, antibiotics, antihistamines, eye solutions for glaucoma management, and retinal eye drops. If shared, the safety concerns of unsupervised usage may range from mild side effects, such as burning or conjunctivitis, to severe adverse reactions, such as toxic epidermal necrolysis and angina pectoris (Latanoprost. IBM Micromedex Solutions, 2021). Sharing of gastroduodenal medications could also lead to serious adverse events (Fossmark et al., 2019). Moreover, some antidiarrheal medications should be used only while following specific patient instructions on dose and treatment duration (Latanoprost. IBM Micromedex Solutions, 2021; Whittaker and Newman, 2021). As findings related to sharing of ophthalmic and gastrointestinal medications have not been reported in detail in other studies, we believe that our study highlights the special role of healthcare professionals, especially pharmacists, in conducting frequent medication reviews and educating their patients to avoid adverse consequences.

Several limitations of this study should be noted. First, our study shares an inherent weakness related to cross-sectional design with limited representativeness and generalizability as a convenience sampling method was used to recruit the survey participants. To improve the external validity and to minimize selection bias, stratification method was used during the recruitment process reflecting the national census statistics by age, gender, and region. In addition, the biggest consumer panel available in South Korea was employed (Kim et al., 2020; Panel Marketing Interactive, 2021). Second, our study included people younger than 70 year old as the study utilized an internet-based survey. The rate of Internet use among Koreans in their 30s was 99.9% and 91.5% for those in their 60s, whereas the rate dropped to 40.3% for those aged 70 and over (National Information Society Agency, 2020). With the very low rate of the internet use among aged 70 and over, we believe that including only those who were younger than 70 was a practical and reasonable approach. Third, as the survey depended on the respondents’ lifetime memory, presence of the measurement bias with underreporting of the medication sharing experience can exist. Finally, although the target medications for the sharing experience was explicitly declared to prescription medications, a possibility of data contamination of their experiences with over-the-counter medications cannot be ignored.

Medication sharing is a multifaceted problem requiring astute observations of patients and persistent efforts by healthcare professionals in the care network. More research is needed to compile data on key characteristics regarding people engaging in medication sharing, types of shared medications, and circumstances surrounding medication sharing. Based on real-world evidence, healthcare professionals could reflect on their patients’ needs for accessing certain medications (e.g., subjective perception of emergency situations and their needs for usage) and be ready to educate and guide them with specific action plans regarding how to appropriately access prescription medications. From the policymakers’ perspective, patient empowerment strategies in the form of public education or campaigns could be considered to improve awareness and avoid the adverse ramifications of unsupervised medication sharing.

## Conclusion

Approximately one in two Korean adults in our study had experience in lending or borrowing medications prescribed for someone else with analgesics as the most frequently shared medications. Respondents with lower educational level were more likely to share ophthalmic medications. Larger scale population based studies are needed to confirm the prevalence of medication sharing and to focus on establishing evidence-based strategies for patient education and improving the medication use process, especially for medications with potential risks when shared without appropriate supervision of healthcare professionals.

## Data Availability

The datasets presented in this article are not readily available because informed consents agreed by the participants indicated that access to the data is limited to the authors and the Institutional Review Board (IRB) of Seoul National University in special conditions. Requests to access the datasets should be directed to EL.
