# Supplementary material for: Evaluation of Prescription Medication Sharing Among Adults in South Korea: A Cross-Sectional Survey
Source: Front Pharmacol. 2022 Feb 4;13:773454. doi: 10.3389/fphar.2022.773454 (PMC8854370; doi:10.3389/fphar.2022.773454)
Supplement: Supplementary file 1 [file DataSheet1.docx]

Supplementary Material

# Supplementary Tables

**Supplementary Table 1. Reasons for lending (*n*=406) or borrowing (*n*=430) prescription medications**

| **Reasons for lending** | N* (%) | **Reasons for borrowing** | N* (%) |
| --- | --- | --- | --- |
| I happened to have the medication the borrower needed. | 237 (58.4%) | The lender had the same (or similar) medication that I needed at the moment. | 195 (45.3%) |
| The borrower was not sick enough to see a doctor. | 156 (38.4%) | I was not sick enough to see a doctor. | 185 (43.0%) |
| The borrower could not see a doctor. | 105 (25.9%) | I was not in a situation to see a doctor. | 100 (23.3%) |
| The borrower urgently needed to use the medication. | 77 (19.0%) | I urgently needed to take the medication. | 73 (17.0%) |
| The borrower wanted stronger medication. | 39 (9.6%) | I trusted the lender. | 47 (10.9%) |
| I just wanted to help the borrower | 29 (7.1%) | I wanted stronger medication. | 34 (7.9%) |
| Total | 643 (158.4%) | Total | 635 (147.7%) |

*Respondents were asked to report reasons for medication lending or borrowing that included up to six factors; thus, the numbers are not mutually exclusive.

**Supplementary Table 2. Factors associated with prescription medication lending experience (*n*=406) by medication type**

| **Participant characteristics** | **Gastroduodenal ulcer medication**  **OR (95% CI)**^a^ | **Ophthalmic medication**  **OR (95% CI)**^a^ | **Antibiotics**  **OR (95% CI)**^a^ | **Hypnotics**  **OR (95% CI)**^a^ |
| --- | --- | --- | --- | --- |
| Gender |  |  |  |  |
| Female | 1.0 | 1.0 | 1.0 | 1.0 |
| Male | 1.32 (0.81–2.15) | 0.88 (0.53–1.45) | 1.07 (0.61–1.90) | 1.03 (0.42–2.51) |
| Age distribution |  |  |  |  |
| 20–29 years | 1.0 | 1.0 | 1.0 | 1.0 |
| 30–39 years | 3.36 (1.26–8.95)* | 1.57 (0.66–3.74) | 4.92 (1.68–14.44)^†^ | 1.30 (0.29–5.83) |
| 40–49 years | 3.43 (1.31–8.99)* | 0.98 (0.40–2.38) | 2.60 (0.85–8.00) | 0.53 (0.10–2.92) |
| 50–59 years | 3.85 (1.42–10.41)^†^ | 0.71 (0.28–1.82) | 3.36 (1.05–10.67)* | 1.19 (0.25–5.80) |
| 60–69 years | 3.64 (1.24–10.70)* | 1.16 (0.43–3.13) | 5.65 (1.65–19.29)^†^ | 0.88 (0.15–5.23) |
| Marital status |  |  |  |  |
| Single | 1.0 | 1.0 | 1.0 | 1.0 |
| Married | 0.75 (0.40–1.40) | 1.04 (0.53–2.04) | 0.40 (0.19–0.81)* | 1.10 (0.33–3.71) |
| Residential area |  |  |  |  |
| Capital | 1.0 | 1.0 | 1.0 | 1.0 |
| Metropolitan cities | 0.93 (0.47–1.86) | 0.82 (0.39–1.72) | 0.55 (0.24–1.24) | 0.94 (0.29–3.11) |
| Provinces | 0.86 (0.48–1.55) | 0.99 (0.53–1.84) | 0.72 (0.37–1.40) | 0.64 (0.22–1.84) |
| Level of education |  |  |  |  |
| High school and below | 1.0 | 1.0 | 1.0 | 1.0 |
| College and above | 1.42 (0.80–2.52) | 0.54 (0.31–0.93)* | 1.37 (0.69–2.73) | 0.72 (0.27–1.91) |
| Health insurance type |  |  |  |  |
| Self–employed | 1.0 | 1.0 | 1.0 | 1.0 |
| Workplace | 0.70 (0.40–1.23) | 1.02 (0.56–1.85) | 1.46 (0.69–3.06) | 0.88 (0.31–2.45) |
| Others^b^ | 1.10 (0.34–3.51) | 0.77 (0.23–2.63) | 2.33 (0.62–8.77) | 0.63 (0.07–5.77) |

^a^Multiple logistic regression analysis.
^b^Others include Medical Aid, no health insurance, or unknown.

**p*<0.05, †*p*<0.01.

*CI* confidence interval, *OR* odds ratio.

**Supplementary Table 3. Factors associated with prescription medication borrowing experience (*n*=430) by medication type**

| **Characteristics** | **Gastroduodenal ulcer medication**  **OR (95% CI)**^a^ | **Ophthalmic medication**  **OR (95% CI)**^a^ | **Antibiotics**  **OR (95% CI)**^a^ | **Hypnotics**  **OR (95% CI)**^a^ |
| --- | --- | --- | --- | --- |
| Gender |  |  |  |  |
| Female | 1.0 | 1.0 | 1.0 | 1.0 |
| Male | 0.86 (0.50–1.47) | 1.25 (0.70–2.21) | 0.91 (0.49–1.67) | 0.25 (0.09–0.69)^†^ |
| Age distribution |  |  |  |  |
| 20–29 years | 1.0 | 1.0 | 1.0 | 1.0 |
| 30–39 years | 2.48 (0.85–7.28) | 1.80 (0.68–4.75) | 3.64 (1.12–11.79)* | 15.89 (1.70–148.34)* |
| 40–49 years | 2.86 (0.97–8.49) | 0.75 (0.25–2.21) | 1.64 (0.46–5.84) | 10.87 (1.08–108.99)* |
| 50–59 years | 3.88 (1.25–12.06)* | 1.21 (0.42–3.50) | 1.97 (0.56–6.99) | 8.16 (0.60–110.07) |
| 60–69 years | 4.34 (1.30–14.49)* | 1.60 (0.52–4.95) | 2.65 (0.70–10.01) | 50.87 (4.34–595.88)^†^ |
| Marital status |  |  |  |  |
| Single | 1.0 | 1.0 | 1.0 | 1.0 |
| Married | 0.59 (0.30–1.17) | 0.86 (0.41–1.80) | 0.97 (0.45–2.11) | 0.14 (0.04–0.47)^†^ |
| Residential area |  |  |  |  |
| Capital | 1.0 | 1.0 | 1.0 | 1.0 |
| Metropolitan cities | 1.11 (0.54–2.30) | 1.04 (0.49–2.22) | 0.28 (0.10–0.76)* | 0.74 (0.18–3.02) |
| Provinces | 0.73 (0.38–1.39) | 0.64 (0.32–1.28) | 0.79 (0.40–1.55) | 1.13 (0.38–3.40) |
| Level of education |  |  |  |  |
| High school and below | 1.0 | 1.0 | 1.0 | 1.0 |
| College and above | 1.58 (0.82–3.07) | 0.66 (0.35–1.25) | 0.63 (0.31–1.27) | 2.61 (0.74–9.13) |
| Health insurance type |  |  |  |  |
| Self–employed | 1.0 | 1.0 | 1.0 | 1.0 |
| Workplace | 1.73 (0.86–3.50) | 1.47 (0.70–3.10) | 1.64 (0.73–3.69) | 0.85 (0.29–2.51) |
| Others^b^ | 1.17 (0.29–4.66) | 1.22 (0.35–4.29) | 1.41 (0.34–5.86) | 0.87 (0.09–8.52) |

^a^Multiple logistic regression analysis.
^b^Others include Medical Aid, no health insurance, or unknown.

**p*<0.05, †*p*<0.01.

*CI* confidence interval, *OR* odds ratio.
